# Rhizosphere microbiome and calcium synergy drive suppression of *Ceratocystis paradoxa* in tropical coconut soils

**DOI:** 10.1007/s00203-026-05108-w

**Published:** 2026-08-18

**Authors:** Kize Alves Almeida, Priscilla de Fátima Pereira, Rafaela Araújo Guimarães, Muhammad Siddique Afridi, Julio Carlos Pereira da Silva, Bruna Andrade, John Quensen, James Tiedje, Jorge Teodoro de Souza, Viviane Talamini, Eudes de Arruda Carvalho, Flávio Henrique Vasconcelos de Medeiros

**Affiliations:** 1 Bayer Crop Science, Sorriso, MT Brazil; 2 Department of Plant Pathology, Universidade Federal de Lavras (UFLA), Lavras, MG 37200-900 Brazil; 3 Department of Plant Health, Universidade Federal de Santa Maria, Santa Maria, RS Brazil; 4 Agrícola Andreis, Av. Barão Do Rio Branco, Cascavel, PR 1597 Brazil; 5 Department of Plant and Soil Science, Michigan State University, 1066 Bogue St., Room, East Lansing, MI A540 USA; 6 Embrapa Tabuleiros Costeiros, Av. Beira Mar, Aracaju, SE 3250 Brazil; 7 Embrapa Recursos Genéticos E Biotecnologia (Cenargen), Parque Estação Biológica, Brasília, DF Brazil

**Keywords:** Suppressive soil, Soil biophysicochemical traits, Microbial communities, Stem bleeding, Soil pH

## Abstract

*Ceratocystis paradoxa* is a polyphagous, soil-borne pathogen that threatens various crops worldwide. This study evaluated soil suppressiveness against *C. paradoxa* and identified key biotic and abiotic factors involved. Suppressiveness was assessed using banana peel baits, followed by comparative analysis of microbial communities and soil physicochemical properties in five suppressive and five conducive soils. Suppressive soils exhibited higher culturable bacterial populations and enrichment of Actinobacteria, Proteobacteria, Firmicutes, and Acidobacteria, particularly Solirubrobacter, Actinoplanes, and Conexibacter. Favorable physicochemical traits included higher pH, calcium content, cation exchange capacity, base saturation, and sand content. Calcium carbonate amendments enhanced suppressiveness, particularly at pH 7.0. Transferring the microbiota from suppressive soils to conducive subsoils, combined with calcium carbonate, also induced suppressiveness. These results highlight the synergistic role of microbial communities and soil chemistry, particularly pH and calcium in pathogen suppression. Calcium promoted microbial activity and stabilized pH, contributing to reduced disease incidence. This integrated strategy offers a promising avenue for sustainable management of *C. paradoxa* in tropical agriculture through microbiome manipulation and soil amendments.

**Supplementary Information:**

The online version contains supplementary material available at https://doi.org/10.1007/s00203-026-05108-w.

## Introduction

*Ceratocystis paradoxa*, a destructive soil-borne pathogen with a polyphagous nature, poses an alarming threat to economically significant crops in tropical and subtropical regions. The pathogen survives in the soil as chlamydospores (Nasution et al. 2019) and infects various economically important crops, such as pineapple (*Ananas comosus* (L.) Merr.), banana (*Musa* spp.), sugarcane (*Saccharum officinarum* L.), and coconut (*Cocos nucifera* L.). Infection by this pathogen commonly results in a disease known as stem bleeding. Notably, a significant proportion of *Ceratocystis* species maintain intrinsic ecological relationships with insects that act as vectors, and *C. paradoxa* belongs to this group (Rahman et al. 2009; Santos et al. 2017; Guzzo et al. 2018; Silva et al. 2023).

The rapid spread of stem bleeding has raised significant concerns regarding its uncontrolled damage to various crops, which are often cultivated in close proximity. Environmental stressors and mechanical damage increase both the spread and colonization by the pathogen, and the most important susceptible crops are frequently subjected to management practices, such as pruning and harvesting, that result in wounds. Furthermore, the absence of resistant cultivars and the limited effectiveness of conventional fungicides present challenges for disease control, particularly in perennial crops (Warwick and Passos et al. 2009; Velásquez et al. 2018).

Within the complex domain of soil biology, the interaction between microbial communities and physicochemical characteristics significantly influences the development of soil suppressiveness against severe soil-borne pathogens (Bonanomi et al. 2010; De Corato 2020). The ability to suppress the growth of soil-borne pathogens can arise from inherent characteristics of the soil or can be induced by a combination of biotic and abiotic factors (Liu and He 2019; Silva et al. 2018).

This phenomenon is underpinned by the intricate dynamics of the soil microbiome, particularly bacterial communities, and key physicochemical traits. For instance, various soil components have been identified as key contributors to pathogen and disease suppression, including soil pH, nutrient levels, texture, organic matter content, and the activity of the microbiome, along with the intricate interactions among these elements (Davey et al. 2019; Muneer et al. 2024). Biotic characteristics have been extensively investigated as key factors contributing to soil suppressiveness against pathogens. These features have the potential to hinder pathogen establishment through multiple mechanisms, such as antibiosis, competition, and other yet-to-be-determined factors (Toju et al. 2018a, 2018b). Nevertheless, soil physicochemical properties can exert a substantial influence on the promotion or inhibition of suppressiveness by modulating the establishment and activities of the microbiome as well as the life cycle of the pathogen (Yuan et al. 2021).

Understanding the soil properties that govern suppressiveness has the potential to facilitate the establishment of sustainable disease management strategies through the stimulation of microbial activity, engineering of the microbiome, and manipulation of soil conditions (Davey et al. 2019; Liu and He 2019; Yuan et al. 2021). Several studies have demonstrated the significant impact of pH on the composition and structure of soil microbiomes, particularly in relation to its detrimental effects on the life cycle of plant pathogens (Gong et al. 2024). Furthermore, nutrients such as calcium influence soil microbes, particularly those involved in the nitrogen cycle, and calcium is frequently applied as lime. In agriculture, calcium is used to reduce aluminum saturation and improve the availability of other nutrients to plants. Beyond nutrient supply, calcium influences soil pH, inducing changes in soil chemistry (Bossolani et al. 2021; Holland et al. 2021).

Hence, an in-depth investigation of the biotic and abiotic characteristics of suppressive soils with respect to *C. paradoxa* is necessary to better understand disease management strategies in areas conducive to disease development. Within this framework, we hypothesized that soil suppressiveness of *C. paradoxa* in coconut soils from Sergipe and Pará, Brazil is influenced by a combination of biological and physicochemical features. The objective of this study was to investigate the biotic and physicochemical characteristics of coconut soils in order to assess their role in the suppressiveness of *C. paradoxa*. Additionally, the study aimed to identify strategies for enhancing suppressiveness in conducive soils.

## Material and methods

### Description of sites and sampling methods

Two coconut production areas affected by stem bleeding disease (*Ceratocystis paradoxa*) were investigated. The first area is located at Platô de Neópolis, site of H. Dantas (S 10.3411°, W 36.7087°), in the state of Sergipe, Brazil. The second area is in Moju (S 2.1017°, W 48.6645°), Pará state, Brazil. Both sites encompass approximately 200 ha of coconut plantations. The variety cultivated at both locations is Anão Verde, with plants spaced 7.5 m apart and grown under a continuous irrigation system. Soil samples were collected from both diseased plants exhibiting stem bleeding symptoms and healthy plants showing no visible symptoms. Samples were taken from five randomly selected points beneath the canopy of healthy plants, at a depth of 0-10 cm, and mixed to form a composite sample of 500 g. A total of 54 composite samples were collected: 25 from Sergipe (labeled T1-T25) and 29 from Pará (labeled T26-T54). One portion of each composite sample was used for soil DNA extraction (stored at -20 °C), while another portion was used for the suppressiveness screening test (stored at 4 °C).

### Soil suppressiveness screening against Ceratocystis paradoxa

To evaluate the disease-suppressive ability of various soil samples against *C. paradoxa*, the pathogen was introduced into the soil, and its recovery was quantified using banana peel baits (Ferreira et al. 2010). Thirty grams of each sampled soil were placed in 90-mm Petri dishes. An 8-mL suspension of the pathogen at a concentration of 1 × 10^5^ conidia mL^-1^ was then applied. The *C. paradoxa* strain TCTL003, obtained from the mycological collection of Embrapa Tabuleiros Costeiros, was used throughout all experimental procedures. The plates were sealed and arranged in a completely randomized design with five replicates, then incubated at 28 ± 2 °C for 7 days. After this incubation period, 13 pieces of banana peel, each measuring 1 cm^2^ and sourced from ripe fruits of the Prata cultivar, were evenly distributed over the surface of the infested soil. The banana peels were surface-sterilized with 1% bleach solution for 30 s, followed by 70% ethanol for 30 s, and finally rinsed with distilled water. The plates were re-sealed and maintained under the same conditions for an additional 7 days, after which the percentage of colonized baits was assessed. Control samples included subsoil classified as Brazilian Latosols (Oxisols) collected from Universidade Federal de Lavras (TS), and coarse construction sand that had never been used to cultivate any plant species (TA). The sand was processed by sieving and sterilization in an autoclave at 121 °C for one hour over three consecutive days.

### Determination of physical, chemical and biological parameters linked to soil suppressiveness

A total of ten soil samples were selected for this assay: five classified as suppressive (T2, T6, T8, T13, T18) and five classified as conducive (T19, T32, T47, T52, T53). The following soil physical parameters were measured: aluminum (Al), potential acidity (H + Al), sum of bases (SB), cation exchange capacity (CEC), effective cation exchange capacity (effective CEC), pH at 7.0 (T), base saturation (V), aluminum saturation (m), percentage of clay, percentage of sand, percentage of silt, and particle density. These measurements were performed following the methods proposed by Carter and Gregorich (2007). Regarding chemical properties, the following were estimated: hydrogen potential in water (pH), available phosphorus (P), potassium (K), calcium (Ca), magnesium (Mg), zinc (Zn), iron (Fe), manganese (Mn), and copper (Cu), following Page (1982).

The biological parameters of each soil involved the quantification of selected microbial populations, including total bacteria, *Pseudomonas* spp., *Pseudomonas fluorescens*, and *Bacillus* spp. These populations were quantified by plating serial dilutions onto agar plates. Samples of 10 g of each soil were placed in Erlenmeyer flasks containing 90 mL of sterile saline solution (0.85% NaCl) and agitated for 30 min at 25 °C. Then, 100-μL aliquots of each 10 × serial dilution were plated in triplicate onto the following media: (1) total bacterial density was determined using Nutrient Agar (Levine 1954) supplemented with 10 mg L^−1^ cycloheximide; (2) *Pseudomonas* spp. and *P. fluorescens* were quantified using King’s B medium (King et al. 1954) supplemented with 10 mg L^−1^ cycloheximide; (3) *Bacillus* spp. populations were determined using ATCC 573 medium (Sneath 1986). Prior to plating on ATCC 573 medium, the dilutions were incubated at 80 °C for 10 min (Johnson et al. 1982). Bacterial populations were evaluated after 48 h of incubation at 25 °C. The total fungal population was assessed using Potato Dextrose Agar (PDA) supplemented with chloramphenicol (2.5 mg L^-1^). For *Trichoderma* spp., the Trichoderma Selective Medium – Lincoln University (TSM-LU) was used, following McLean et al. (2005). Total fungi were evaluated after five days, and *Trichoderma* spp. after seven days of incubation at 20 °C in the dark.

Each microbial population was quantified by calculating the average number of colonies expressed as colony-forming units per gram of soil (CFU g^-1^), using the formula: CFU g^-1^ = N × 10 × F × Y, where N is the number of colonies, F = 10 (correction factor for plating 100 μL per plate), and Y is the sample dilution factor. For statistical analysis, data were transformed to log (x + 1), where x corresponds to the number of CFU.

### Soil DNA extraction, sequencing, and bioinformatics analysis

The two most contrasting soil samples, one suppressive (T2) and one conducive (T19) were selected for DNA extraction and metataxonomic analysis. The primer pair Bakt341F and Bakt805R, which specifically targets the highly variable V3-V4 region of the 16S rRNA gene, was used. Genomic DNA was extracted from 0.5 g of soil using the DNeasy PowerSoil Kit (Qiagen Inc.) according to the manufacturer’s instructions. Nucleic acid quantification was performed with the dsDNA HS Assay Kit (Invitrogen) on a Qubit 2.0 Fluorometer (Life Technologies, Grand Island, NY). DNA samples were sent to Psomagen Inc. (Rockville, Maryland, USA). Libraries were prepared according to Illumina’s protocol and analyzed using the V3 kit for the Illumina MiSeq platform (2 × 300 bp). Sequences were trimmed of primers using Cutadapt (Martin 2011) and truncated at 244-233 and 250-217 for forward and reverse reads, for the first and second datasets, respectively, using FIGARO (Weinstein et al. 2019). Paired-end reads were merged using USEARCH and processed with QIIME2 (Bolyen et al. 2019) and the DADA2 plugin (Callahan et al. 2016). Operational taxonomic unit (OTU) classification was performed using the RDP classifier (Wang et al. 2007).

### Influence of calcium application on suppressiveness

Three calcium sources were applied to the soils to evaluate their impact on disease suppression: calcium carbonate (CaCO_3_), calcium sulfate (CaSO_4_), and calcium chloride (CaCl_2_). These sources were applied at various concentrations ranging from 1 to 3.5 cmol_c_ dm^-3^, to simulate the calcium concentrations observed in suppressive samples. CaCO_3_ and CaSO_4_ were prepared 50 days prior to the experiment to allow sufficient time for reactions within the soil. Subsoil (TS) was used as the substrate for all treatments. A completely randomized experiment was designed with five replicates, using the banana bait scheme detailed in Sect. 2.2. Data were submitted to non-linear regression according to their best fit. The resulting pH, percentage of colonized baits, and number of perithecia of *C. paradoxa* were evaluated. Biotic and abiotic properties were then correlated using Pearson’s correlation coefficient.

### Suppressiveness transfer

The suppressiveness transfer was assessed by extracting microbial communities from suppressive soil T2 and inoculating them into autoclaved subsoil (TS), previously classified as conducive. A 10% dilution (10 g of soil in 90 mL of sterile 0.85% NaCl solution) was blended at high speed for 60 s, and the supernatant was passed through a 5-μm sieve to remove remaining particles or debris. The microbiome of suppressive soil T2 was pelleted for 10 min at 4000 × g and resuspended in 60 mL of sterile ultrapure water (Elhady et al. 2018). Subsequently, plates containing 30 g of TS were treated with a 4-mL suspension of soil microbiota, equivalent to 70% of the soil’s water-holding capacity. The transplanted microbiomes were allowed to establish for seven days, after which the pathogen was added at a concentration of 10^5^ conidia mL^-1^ and maintained for an additional seven days. Banana peels were placed on the soil surface as baits for pathogen colonization, as described in Sect. 2.2. Some plates with TS soil received calcium carbonate (1 mg dm^-3^) on the soil surface 50 days before the experiment was installed, and were maintained at 60% of soil water-holding capacity to facilitate interactions between calcium and the soil. Treatments included: (a) extracted suppressive soil microbiome (T2); (b) calcium carbonate; (c) combined extracted suppressive microbiome with calcium carbonate; (d) subsoil (TS); and (e) 30 g of suppressive soil (T2).

### Statistical analyses

Statistical analyses were conducted using R software version 3.6.1 (https://www.R-project.org/). Prior to analysis, data were subjected to normality tests (Shapiro-Wilk) and tests for homogeneity of error variance (Bartlett). Data were transformed where necessary. Analysis of variance (ANOVA) was performed, and means were compared using the Scott-Knott test (P < 0.05). Soil biophysicochemical properties were analyzed using a correlation matrix and principal component analysis (PCA). Spearman’s rank correlation, a non-parametric method robust to non-normal data was employed, with results visualized using ggcorrplot (displayed as a lower triangle matrix, excluding non-significant correlations at α ≤ 0.1). This more permissive threshold was adopted specifically for the exploratory correlation matrix, given the limited sample size (n = 10 soils), and is distinct from the p < 0.05 threshold used for the ANOVA and mean-comparison analyses described above. Diversity analysis was performed using the Community Ecology Package (vegan version 2.6-4) ("Community Ecology Package" 2022). Computations were performed using resources from the High-Performance Computing Center (HPCC), Michigan State University, USA. Percentage of colonized baits, perithecia number, and the resulting pH were grouped and analyzed as described above.

## Results

### Soil suppressiveness in coconut plantation areas

**Fig. 1:**
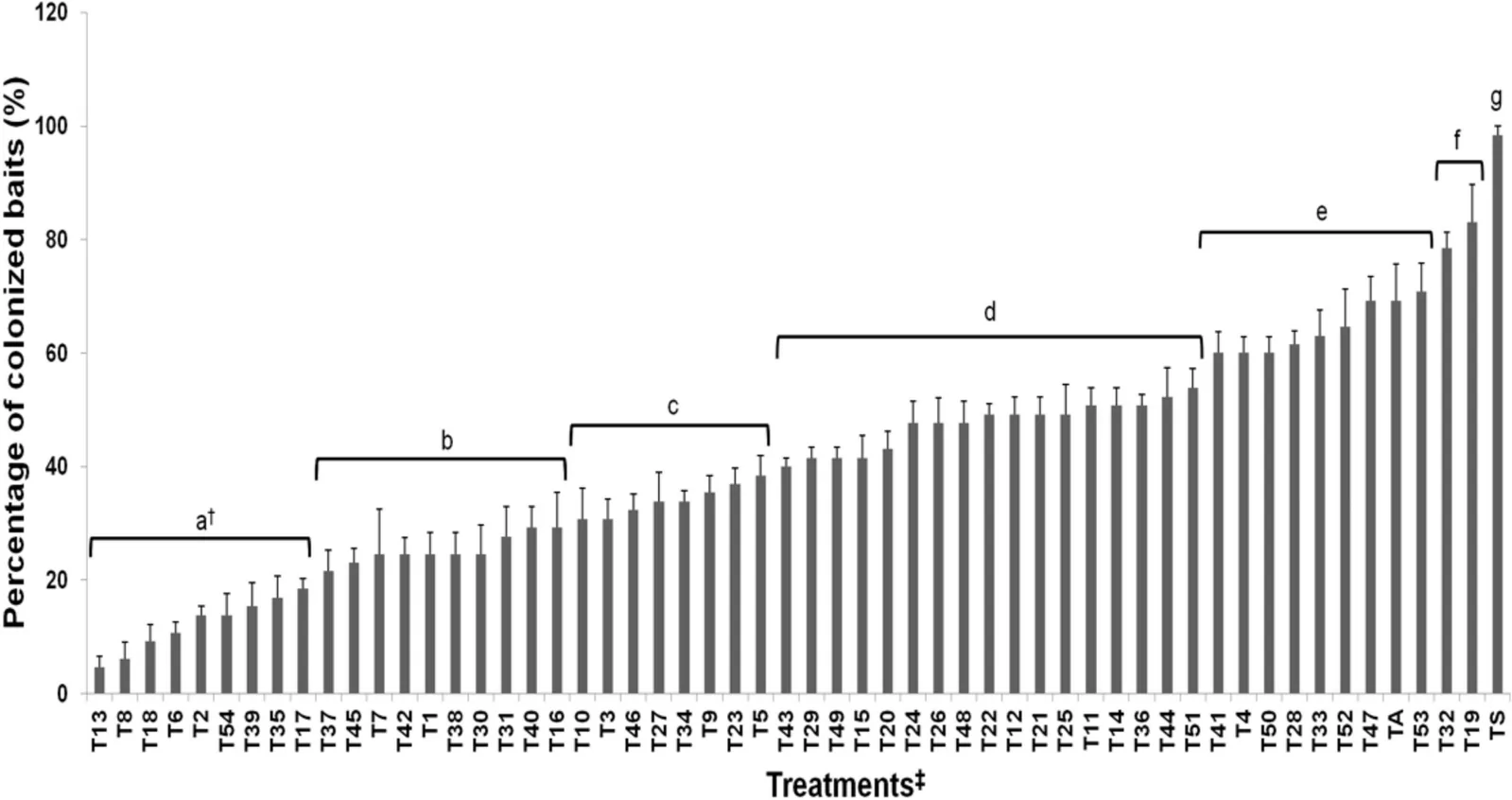
Percentage of banana peel bait colonization by *Ceratocystis paradoxa*. Means followed by the same letter are not significantly different according to Scott–Knott’s test at a 5% significance level. ‡ Treatments T1-T25 and T26-T54 were collected from Sergipe and Pará, Brazil, respectively. The controls, TA (sand control) and TS (subsoil), were obtained from Lavras, Minas Gerais, Brazil

The colonization rate of *C. paradoxa* across the 56 soil samples (including controls TS and TA) ranged from 4.6% to 98.5% (Fig. 1). Analysis of the number of colonized baits identified seven significantly different groups of soils (p < 0.01). Groups labeled with letters e, f, and g were considered conducive to colonization, while the group represented by letter a was identified as the most suppressive. This suppressive group included samples T13, T8, T18, T6, T2, T54, T39, T35, and T17. Furthermore, soil suppressiveness was not linked to specific geographical locations where the samples were collected, but rather to the colonization ability of the baits.

### Major factors associated with soil suppressiveness

**Fig. 2:**
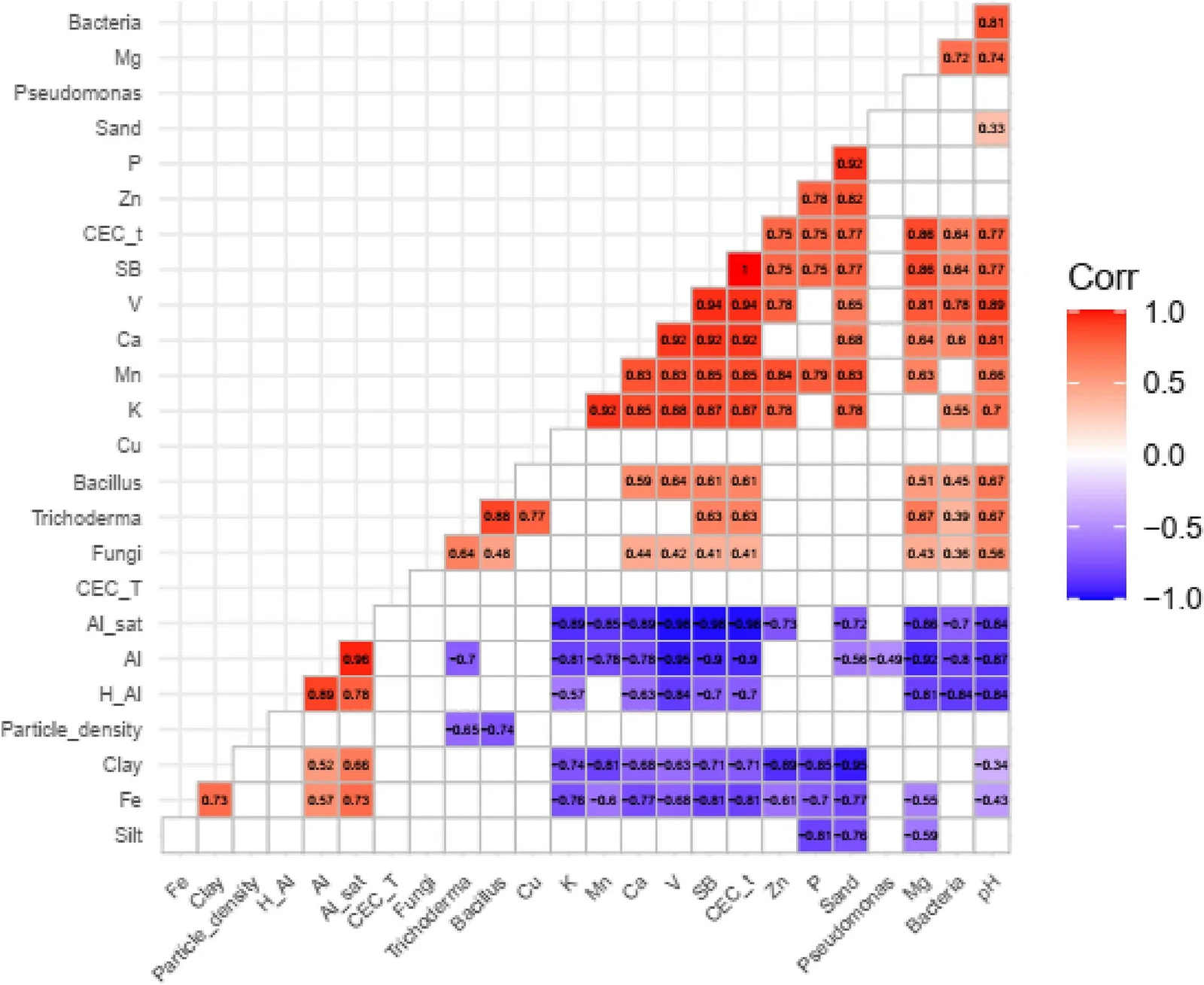
Correlation matrix depicting relationships among physical, chemical, and biological variables in soil samples. The color gradient represents the strength and direction of correlations, ranging from strong negative (blue) to strong positive (red). Key variables include soil nutrients, microbial communities, and soil physical properties

A total of ten soil samples were selected for this assay, five suppressive (T2, T6, T8, T13, T18) and five conducive (T19, T32, T47, T52, T53). Soil biophysicochemical properties correlated with bait colonization are shown in Fig. 2. Positive correlations are indicated in red and negative correlations in blue. Only correlations with α ≤ 0.1 are displayed (an exploratory threshold distinct from the p < 0.05 criterion used for mean comparisons). In total, 13 factors were found to correlate significantly with the analyzed parameters (p < 0.05), including six variables of chemical origin, five of physical origin, and two of biological origin.

Among the chemical variables correlated with bait colonization, Ca showed a positive correlation with Bacillus community density (r = 0.59), K (r = 0.85), Mn (r = 0.83), and Fe (r = 0.73). Mg correlated positively with *Trichoderma* spp. (r = 0.67), Ca (r = 0.64), V (r = 0.81), and SB (r = 0.86). Mn correlated only with K (r = 0.92). P correlated with Mn (r = 0.79) and Zn (r = 0.78). Zn correlated with K (r = 0.78) and Mn (r = 0.84). The chemical factor pH was most broadly correlated, showing positive associations with total fungi (r = 0.56), total bacteria (r = 0.81), *Bacillus* spp. (r = 0.67), K (r = 0.70), Mn (r = 0.66), Ca (r = 0.81), V (r = 0.89), SB (r = 0.77), CEC_t_ (r = 0.77), and Mg (r = 0.74) (Fig. 2).

Among the physical variables, Al content showed positive associations with Fe (r = 0.57), clay (r = 0.52), and H + Al (r = 0.89). Al saturation was positively correlated with Fe (r = 0.76), clay (r = 0.66), H + Al (r = 0.78), and Al (r = 0.96). CEC_t_ was positively correlated with K (r = 0.87), Mn (r = 0.85), Ca (r = 0.92), V (r = 0.94), and SB (r = 1.0). SB correlated with K (r = 0.87), Mn (r = 0.85), Ca (r = 0.92), and V (r = 0.94). Sand content (%) showed positive correlations with Mn (r = 0.83), Ca (r = 0.68), SB (r = 0.77), CEC_t_ (r = 0.77), Zn (r = 0.82), and P (r = 0.92) (Fig. 2).

Among the biological variables, *Bacillus* spp. showed a positive and significant correlation with *Trichoderma* spp. (r = 0.88), while total bacteria correlated positively with Ca (r = 0.60), V (r = 0.78), SB (r = 0.64), CEC_t_ (r = 0.64), and Mg (r = 0.72) (Fig. 2).

### PCA of soil chemical properties: trends differentiating suppressive and non-suppressive soils

**Fig. 3:**
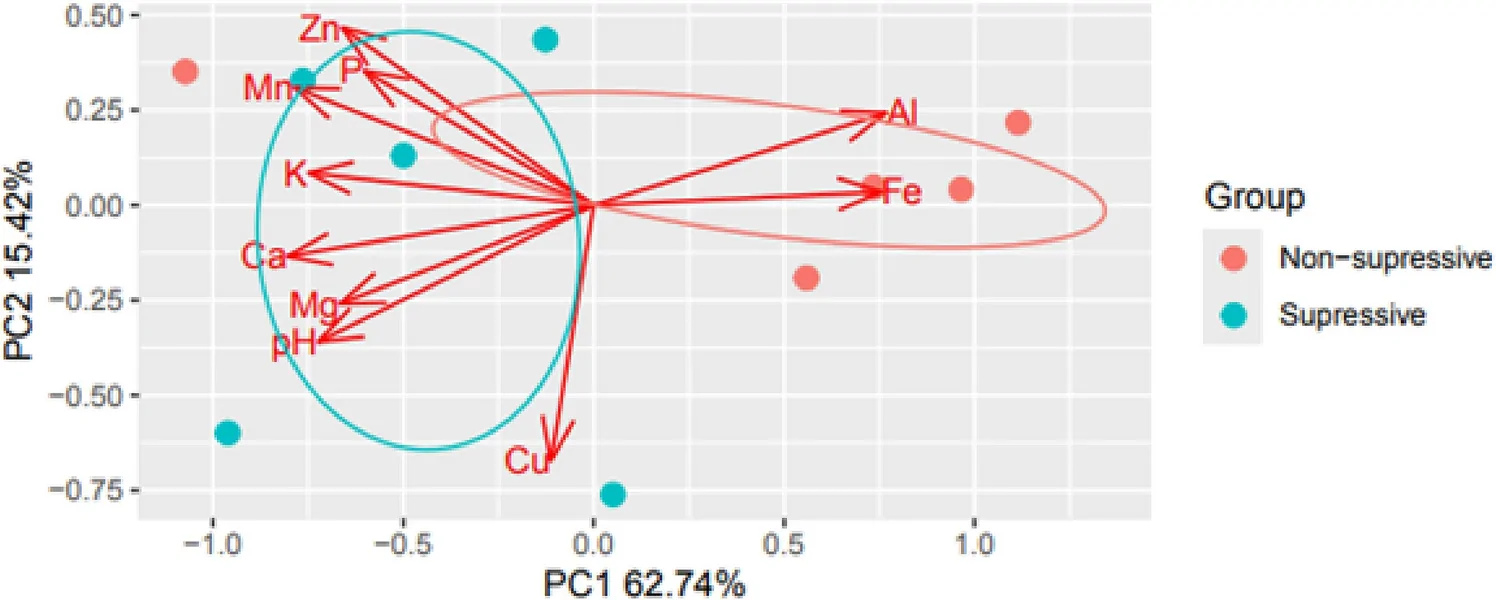
Principal Component Analysis (PCA) of soil chemical properties associated with suppressive and non-suppressive soils in coconut production systems. The biplot displays the distribution of samples (dots) based on their chemical profiles, with red representing non-suppressive soils and blue representing suppressive soils. Arrows indicate the direction and magnitude of each chemical variable’s contribution to the principal components

Regarding the PCA of soil chemical properties (Fig. 3), the first two principal components explained 78.16% of the total variance, with PC1 accounting for 62.74% and PC2 for 15.42%. The biplot indicates a clear separation between the two soil groups: suppressive soils were characterized by higher levels of pH, calcium (Ca), magnesium (Mg), potassium (K), phosphorus (P), manganese (Mn), zinc (Zn), and copper (Cu), whereas non-suppressive soils showed strong associations with higher concentrations of aluminum (Al) and iron (Fe). However, this variation was not statistically significant (p = 0.065). Iron showed a strong positive projection on the first axis and was correlated with Al; both of these variables tended to be negatively correlated with all other chemical variables, with the exception of Cu (Fig. 3).

### PCA of soil physical properties: clay and sand fractions exhibit opposing trends in coconut cultivation

PCA of the physical components of coconut soils revealed a clear correlation between suppressive and non-suppressive group components (p = 0.025). The first two principal components collectively explained 78.30% of the total variance, with PC1 contributing 62.62% and PC2 accounting for 15.68%. The analysis revealed that clay content exhibited higher values in non-suppressive soils, whereas sand demonstrated a strong negative correlation with clay,showed a more pronounced association with cation exchange capacity (CEC_t_) and sum of bases (SB), both of which displayed elevated levels in suppressive soil samples.

### Actinobacteria and Firmicutes enrichment mediates suppressive soil activity against C. paradoxa colonization

The PCA of biological parameters did not show significant cluster separation (PERMANOVA, p = 0.164; Supplementary Fig. S1). However, culturable fungi, *Trichoderma* spp., and *Bacillus* spp. were found to negatively correlate with bait colonization by C. paradoxa (r =  − 0.72, p < 0.01).

**Fig. 4:**
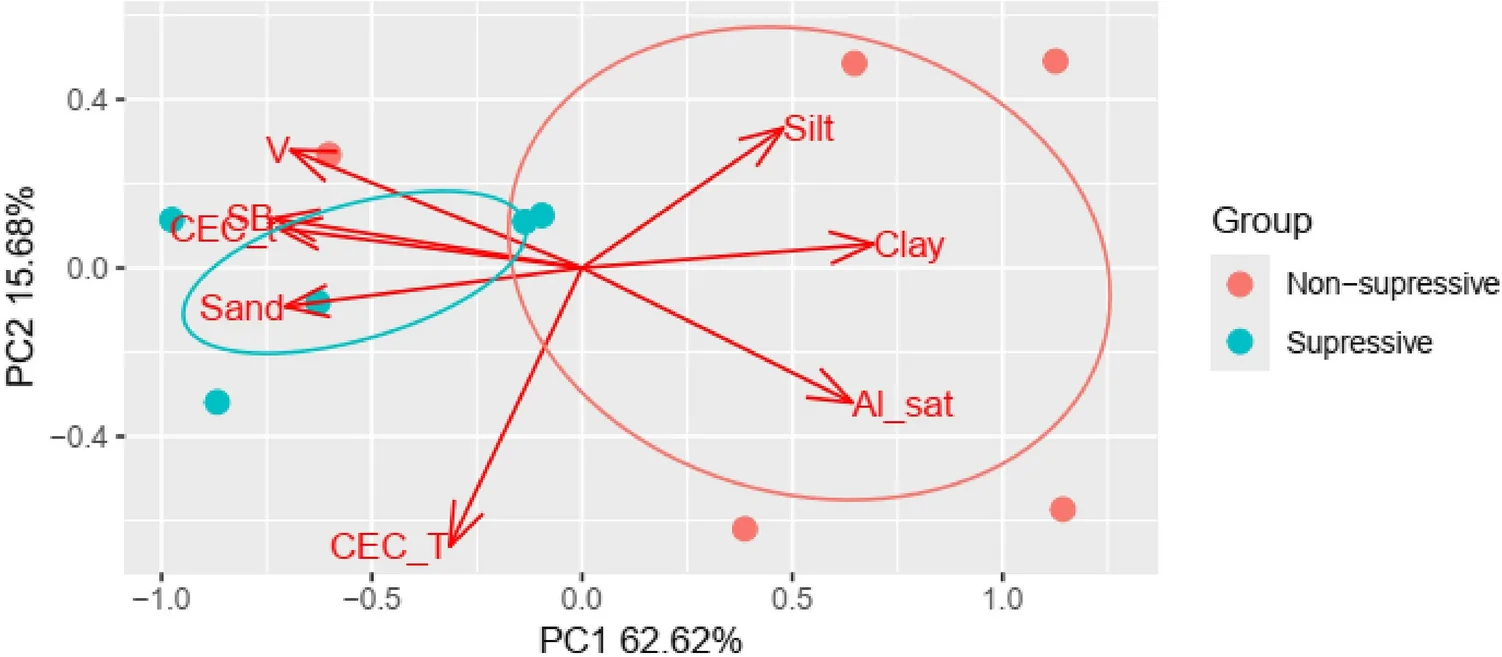
Principal Component Analysis (PCA) of soil physical properties associated with suppressive and non-suppressive soils in coconut production systems. The biplot displays the distribution of samples (dots) based on their physical profiles, with red representing non-suppressive soils and blue representing suppressive soils. Arrows indicate the direction and magnitude of each physical variable’s contribution to the principal components

The taxonomic profile of contrasting soils revealed Actinobacteria, Proteobacteria, Firmicutes, and Acidobacteria as the major phyla in the top ten, corresponding to 80.7% and 82.2% for conducive and suppressive soils, respectively (Fig. 4). Actinobacteria was the most abundant phylum in suppressive soils, corresponding to 28.6% compared to 22.4% in conducive soils. Other representatives included Chloroflexi, Planctomycetes, Verrucomicrobia, Bacteroidetes, Gemmatimonadetes, and WPS-1 (Supplementary Fig. S2).

**Fig. 5:**
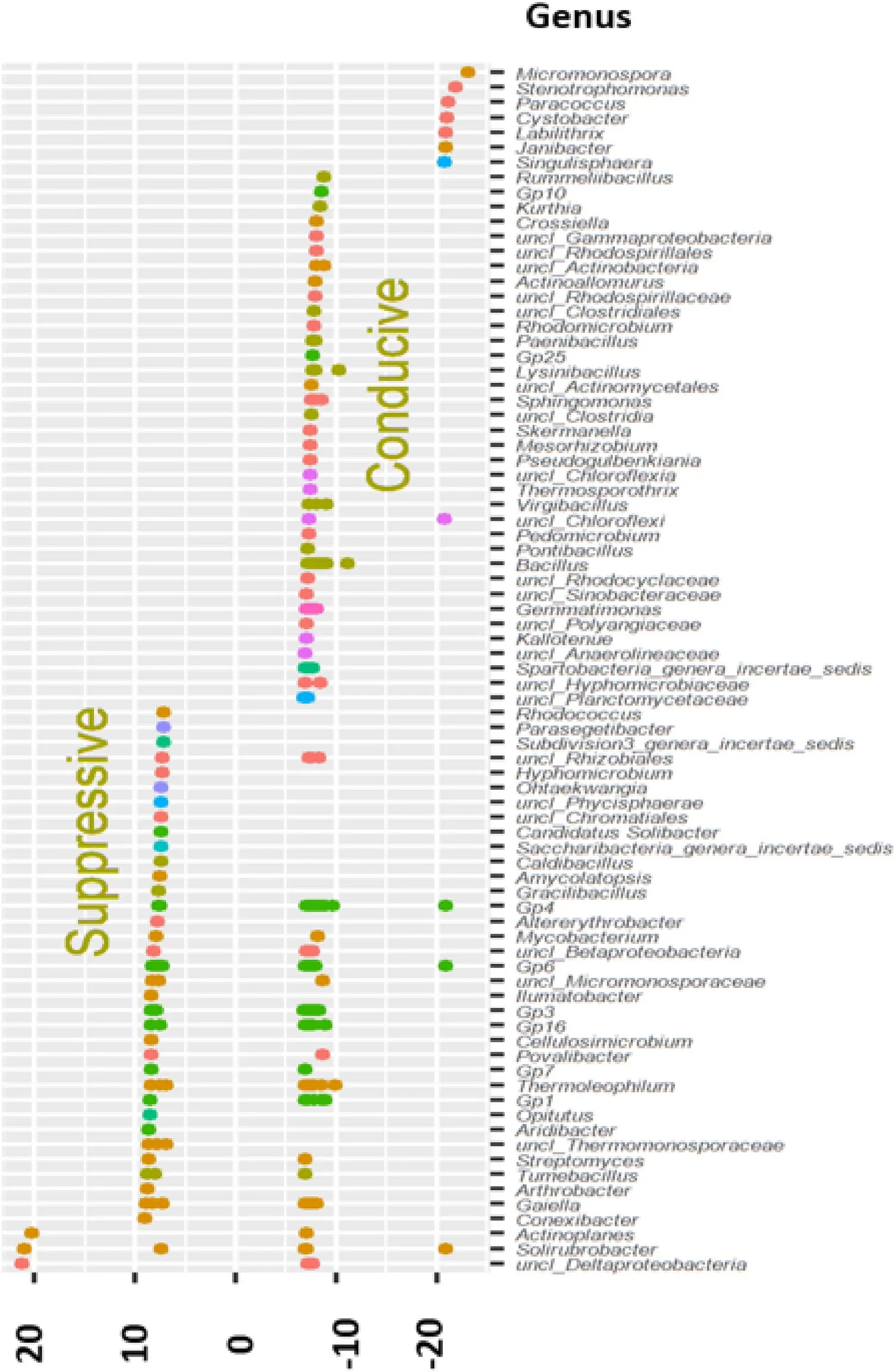
Differential analysis of bacterial communities in suppressive and conducive soils conducted using the DESeq2 package, with data transformed to log_2_ fold change. The analysis highlights shifts in bacterial taxa associated with soil suppressiveness or conduciveness. Log_2_ fold changes are color-coded by genus to facilitate visualization and comparison across different taxonomic levels

Actinobacteria were more abundant in suppressive soils (28.6% vs. 22.4%; DESeq2, q = 0.02, log_2_FC + 1.3), while Firmicutes showed a notably higher abundance of Bacillus spp. in suppressive soils (7.1 times greater, q < 0.001). In contrast, Proteobacteria dominated in conducive soils (31.2% vs. 25.8%) (Supplementary Fig. S2). Differential abundance analysis (ANCOM-BC) identified 212 distinct amplicon sequence variants (ASVs) (False Discovery Rate < 0.05). Suppressive soils showed enrichment of chitinolytic taxa such as *Streptomyces* (log_2_FC + 2.1) and biocontrol taxa such as *Paenibacillus* (log_2_FC + 3.4). These findings indicate that functional guilds of Actinobacteria and Firmicutes are key mediators of soil suppressiveness, highlighting potential targets for microbiome-based disease management in tropical agroecosystems (Fig. 5).

### Calcium source and dose effects on soil pH and suppression of Ceratocystis paradoxa

**Fig. 6:**
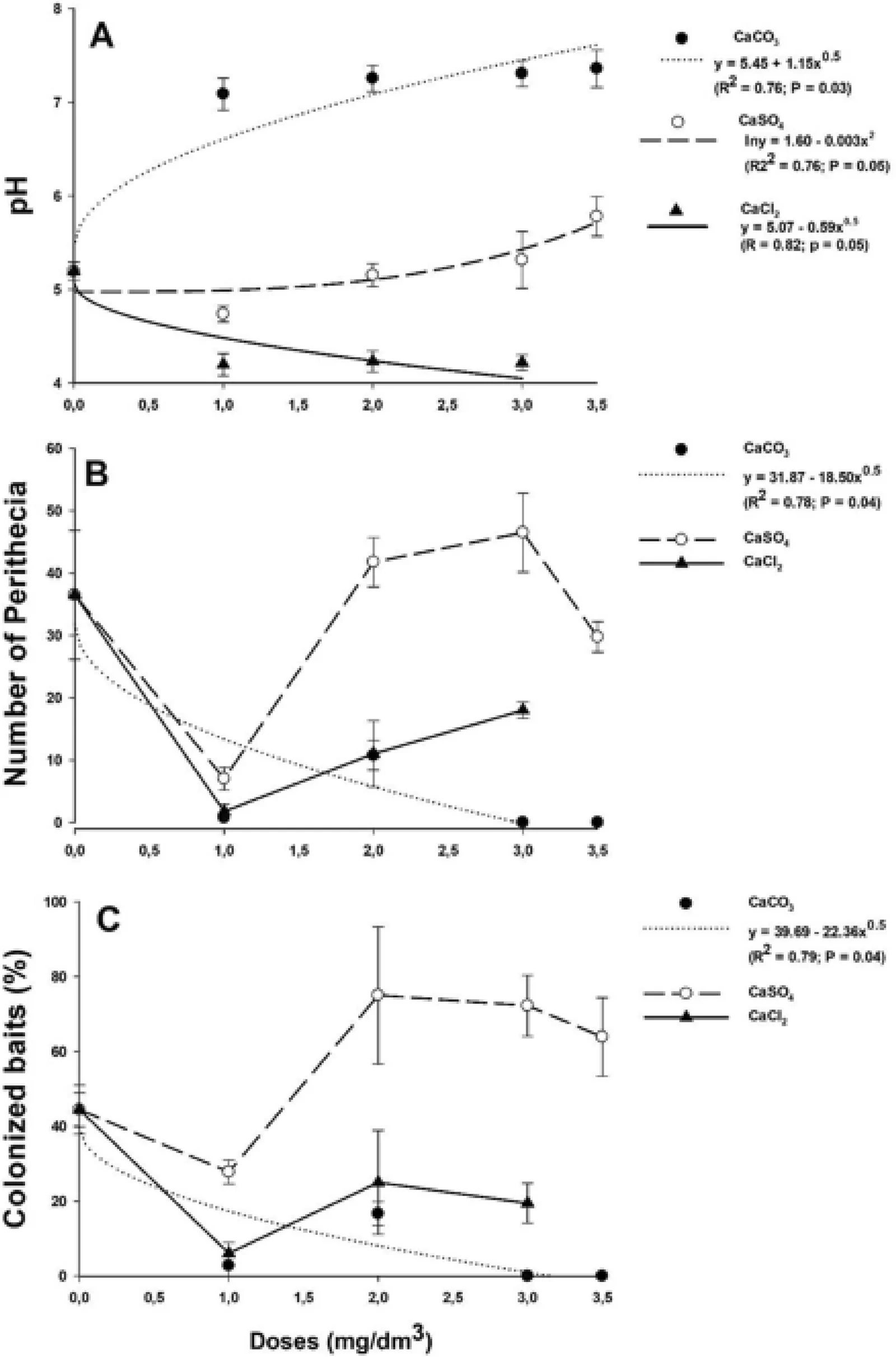
Effect of calcium sources and doses on **A** soil pH, **B** percentage of bait colonization, and (C) number of perithecia. Calcium sources tested were calcium carbonate (CaCO_3_), calcium sulfate (CaSO_4_), and calcium chloride (CaCl_2_), with doses ranging from 0 to 3.5 cmol_c_ dm^-3^. Significant regression trends are indicated

Soil pH showed a significant increase with rising doses when soils were supplemented with different calcium sources (Fig. 6A). The addition of calcium carbonate raised the pH from 5.20 in the control group to 7.09 at the first dose, and further increased it to 7.36 at a concentration of 3.5 cmol_c_ dm^-3^ (p = 0.03). Conversely, calcium sulfate caused a decrease in pH after the first dose, recording a pH of 4.73, followed by an average pH of 5.41 for subsequent concentrations (p = 0.05; Fig. 6A). For all four doses of calcium chloride, the average pH was 4.21 (p = 0.05). The initial dose of calcium carbonate (1 cmol_c_ dm^-3^) led to a 95.89% reduction in the number of perithecia of C. paradoxa compared to the control (p = 0.04; Fig. 6B). Furthermore, application of calcium carbonate at 1 cmol_c_ dm^-3^ reduced bait colonization by 41% compared to the control. When doses of 3 and 3.5 cmol_c_ dm^-3^ were used, bait colonization dropped to just 1% (p = 0.04; Fig. 6C). Increasing doses of calcium sulfate or calcium chloride did not affect colonization rates.

Pearson’s correlation coefficient indicated a strong negative relationship between pH and both bait colonization (r =  − 0.87) and the number of perithecia (r =  − 0.80). In contrast, a clear positive correlation was observed with calcium chloride application, yielding correlation coefficients of r = 0.76 for bait colonization and r = 0.66 for perithecia counts.

### Soil microbiome vs. calcium carbonate: suppression of pathogen perithecia

**Fig. 7:**
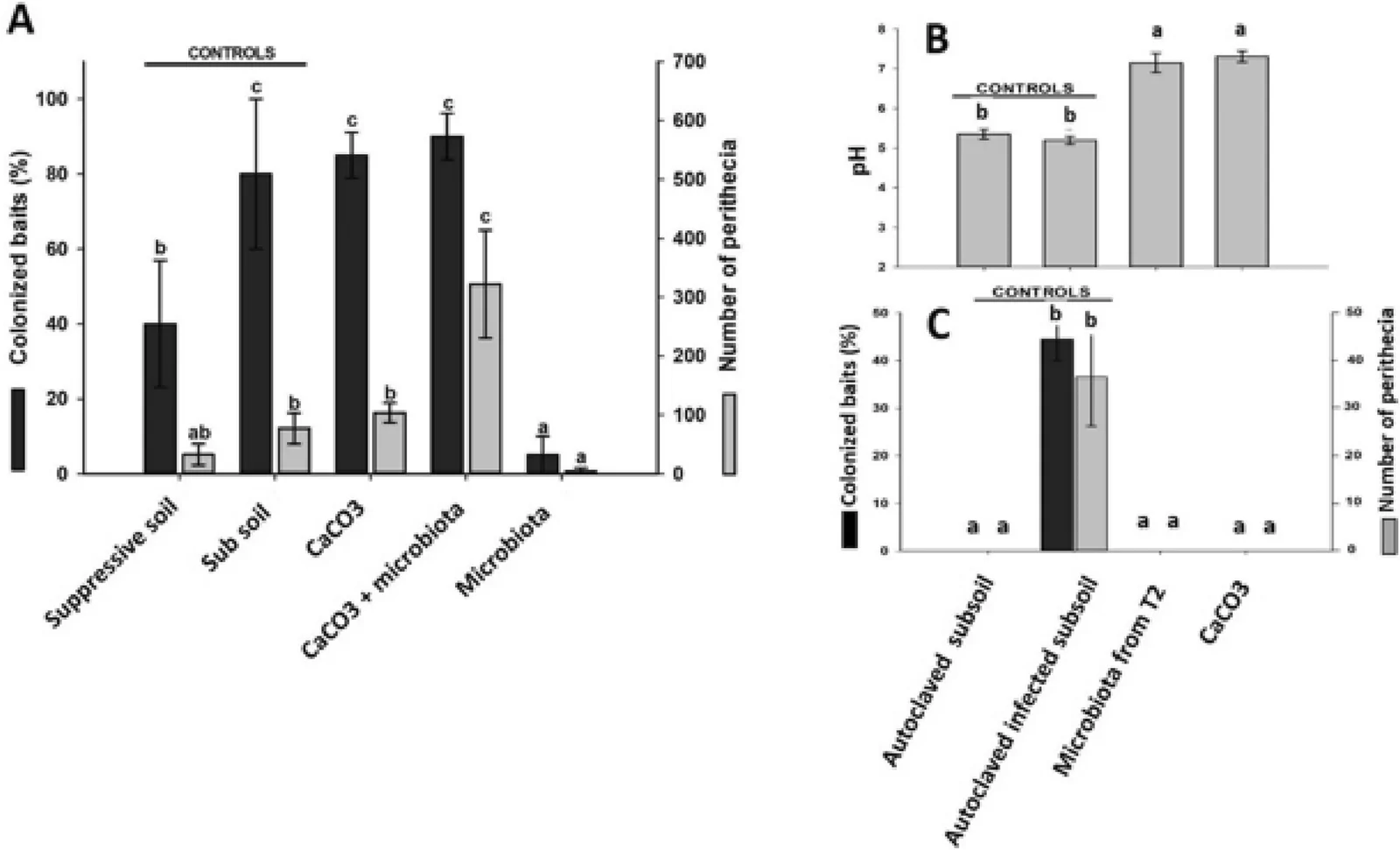
Effect of calcium carbonate (1 mg dm^−3^) and extracted soil microbiota on *Ceratocystis paradoxa* infection and perithecia formation (**A**). The study assessed the impact of suppressive soil (T2) and extracted microbiota (from soil T2), compared to control subsoil (TS), on disease parameters. The effect of calcium carbonate on soil pH (**B**) and on colonization and perithecia number (**C**) was also evaluated. Control conditions included autoclaved subsoil with and without pathogen inoculation. Means followed by the same letter do not differ significantly (p < 0.05) according to Scott–Knott’s test

The extracted suppressive soil microbiome (T2) resulted in significantly lower bait colonization and fewer perithecia than the calcium carbonate treatment alone, applied at a concentration of 1 mg dm^-3^ (~ 80% lower bait colonization and 95.37% fewer perithecia than calcium carbonate alone; p < 0.05; Fig. 7A). The number of perithecia did not differ significantly between calcium carbonate and the control (TS subsoil). However, calcium carbonate perithecia counts were 68.5% lower than those of the original T2 soil (whole suppressive soil, as opposed to the extracted T2-derived microbiome; Fig. 7A). Suppressive soil (T2) and the extracted microbiome showed a reduction of up to 93.8% in the number of pathogen structures (perithecia) compared to the control TS. There was no significant difference between the suppressive soil and the extracted microbiome, although the original soil had a slightly greater number of perithecia. Interestingly, when calcium carbonate and extracted soil microbiota were combined, the number of perithecia increased significantly (Fig. 7A). Soil pH tended toward neutral (7.0) when calcium was added (Fig. 7B and C).

## Discussion

In this study, biotic properties were among the key drivers of the suppressive phenotype (< 20% bait colonization) against *C. paradoxa*. Seven of the ten evaluated soils exhibited significantly increased pathogen growth (bait colonization) after sterilization. Although total fungi, *Bacillus*, *Pseudomonas*, and *Trichoderma* were not significantly different between conducive and suppressive soils, total bacteria were higher in suppressive soils.

The observed variation in soil suppressiveness within coconut plantation regions can be attributed to microbial communities and plant–microbe interactions. Soils exhibiting high suppressiveness harbor antagonistic bacteria that hinder the growth and reproduction of *C. paradoxa* (Durán et al. 2018; Ros et al. 2017). In contrast, conducive soils may provide favorable conditions for the development and proliferation of this disease, which can be affected by variables such as nutrient availability, pH, moisture content, and organic matter concentration (Griffin 2012).

The differences in soil properties between suppressive and conducive soils provide insight into the factors that influence suppressiveness in coconut cultivation areas. Soils with high pH, calcium levels, sum of bases, and base saturation are suppressive, creating unfavorable conditions for pathogen growth by affecting nutrient availability and altering the composition of microbial communities (Postma et al. 2008). Their elevated effective cation exchange capacity (CEC) improves nutrient retention and promotes microbial activity. In addition, higher sand content enhances drainage and aeration, thereby impeding pathogen spread. Conversely, soils favorable for pathogen survival are those with high levels of iron and clay, as these soils retain more moisture and have less oxygen available (Bongiorno et al. 2019; Sánchez-Moreno and Ferris 2007).

Furthermore, the conversion of a conducive soil to a suppressive one through microbiome transfer confirms the biotic role in suppressiveness (Fig. 3). Many studies have reported that the suppressive microbiome can be transferred to other soils (Elhady et al. 2018). By manipulating pH levels, we also observed alterations in suppressiveness against *C. paradoxa*, indicating the direct or indirect involvement of physicochemical properties in suppressiveness. Physicochemical changes may interfere with microbiome activity or composition, resulting in significant alterations in suppressive activity against plant pathogens (Vida et al. 2020).

Among the 25 soil properties compared in this study, 52% showed significant differences between the most contrasting soils, and 84.4% of these corresponded to physicochemical characteristics. Although we considered the means of variables across a group of soils (suppressive and conducive), we recognize the limitations of this approach, since suppressiveness in each soil may be conferred by different soil properties. Among the 15 physicochemical properties evaluated, seven differed between suppressive and conducive soils. Soil pH, which was higher in suppressive soils, is involved in the availability of several nutrients and may favor the establishment of antagonistic bacteria (Liu et al. 2019). Additionally, pH may have contributed to the higher levels of calcium, sum of bases, effective CEC, and base saturation, all of which are linked to high fertility. The availability of nutrients may have favored the establishment of selected groups of microorganisms with antagonistic activity against the pathogen (Afridi et al. 2022a; Schlatter et al. 2017). Iron and aluminum levels, however, were higher in conducive soils. Lower iron content selects siderophore-producing microorganisms that compete with soil-borne pathogens for this element (Pelzer et al. 2011; Schlatter et al. 2017). Such mechanisms have been described in different suppressive soils (Afridi et al. 2022a, 2022b) and may also play a role against *C. paradoxa*. Aluminum is toxic to several plants and microorganisms (Kochian et al. 2015) and may be involved in the lower populations of total culturable bacteria in conducive soils. Furthermore, all suppressive soils had significantly higher sand (86.80% vs. 71.40%) and lower clay (7.20% vs. 21.20%) contents, which has also been reported to correlate with suppressiveness to *Fusarium* sp. (Akber et al. 2023; Expósito et al. 2017; Shen et al. 2022).

Calcium can potentially interfere with most of the chemical attributes that differed significantly between groups. Therefore, we tested the hypothesis that calcium is somehow involved in suppressiveness (Saharan et al. 2021). Lime and gypsum, calcium carbonate (CaCO_3_) and calcium sulfate (CaSO_4_) are widely used in agriculture to correct acidic pH by controlling aluminum saturation (Shruthi et al. 2024). Adding calcium carbonate increased suppressiveness against *C. paradoxa*, along with raising the pH level. Specifically, a pH of 7.0 demonstrated the highest efficacy. In contrast, combining calcium carbonate with the extracted suppressive microbiome did not enhance, and in fact reduced, the suppression achieved by the microbiome alone, resulting in a significant increase in perithecia formation relative to the microbiome-only treatment. This suggests a possible antagonistic interaction between calcium carbonate and the transferred microbial community under these experimental conditions, possibly related to pH-driven shifts in microbial activity or composition during the establishment period (Martin et al. 2012; Bossolani et al. 2021). Additionally, antagonists from Actinobacteria, which were more abundant in the suppressive soil, typically perform better against plant pathogens at pH values close to neutral (El-Tarabily and Sivasithamparam 2006; Silva et al. 2018).

This study does not identify a single soil property as the primary driver of suppressiveness. Instead, soil microbiome composition, calcium carbonate application, and pH were highlighted as major drivers of suppression of *C. paradoxa* in soils from coconut farms. Although we could not distinguish among them individually, a negative correlation was observed between pH and bait infection when calcium carbonate was applied. Pathogen growth at pH 7.0 (Fig. 7B) was reduced by almost 100%. Nevertheless, suppressive soils presented higher densities of total bacteria compared to conducive ones. Metataxonomic analysis confirmed that many antagonistic bacterial groups were more abundant in suppressive soils. Calcium carbonate appears to improve microbiome activity, which may be explained by its effect on pH. Therefore, suppressiveness may be induced in conducive soils through calcium applications that act either directly against the pathogen or by promoting the build-up of antagonistic bacteria that control the pathogen in the soil,a key stage in the *C. paradoxa* life cycle before plant infection.

## Conclusion

This study demonstrates that soil suppressiveness against *C. paradoxa* in coconut plantations is determined by an interplay of chemical, physical, and biological factors. Suppressive soils were characterized by higher pH (neutral to slightly alkaline), elevated cation exchange capacity (CEC), and higher bacterial diversity. Microbial analysis revealed that Actinobacteria (e.g., *Streptomyces*) and Firmicutes (e.g., *Bacillus*) were significantly enriched in suppressive soils, along with beneficial fungi such as *Trichoderma*, which correlated with reduced pathogen colonization. Calcium carbonate (CaCO_3_) amendments effectively suppressed *C. paradoxa* by raising soil pH, achieving up to 99% reduction in pathogen activity. These findings suggest that integrating liming practices with microbial bioaugmentation could offer a sustainable strategy for disease management in coconut cultivation, reducing reliance on chemical fungicides while enhancing soil health. Future research should focus on field validation and optimization of these approaches for large-scale application.

## Supplementary Information

Below is the link to the electronic supplementary material.

Supplementary file1 (DOCX 99 KB)

## Data Availability

The datasets generated and/or analyzed during this study are available from the corresponding author upon reasonable request. The raw sequence data of this study have been deposited in the NCBI public repository under BioProject PRJNA1122864.

## Declarations

### Use generative AI or AI-assisted technology

During the preparation of this work the author(s) used Skywork in order to improve and edit English. After using this tool/service, the author(s) reviewed and edited the content as needed and takes full responsibility for the content of the published article.
