# Supplementary material for: Rhizosphere microbiome and calcium synergy drive suppression of Ceratocystis paradoxa in tropical coconut soils
Source: Arch Microbiol. 2026 Aug 18;208(11):586. doi: 10.1007/s00203-026-05108-w (PMC13486078; doi:10.1007/s00203-026-05108-w)
Supplement: Supplementary file 1 [file 203_2026_5108_MOESM1_ESM.docx]

**Rhizosphere microbiome and calcium synergy drive suppression of *Ceratocystis paradoxa* in tropical coconut soils**

Kize Alves Almeida¹, Priscilla de Fátima Pereira², Rafaela Araújo Guimarães², Muhammad Siddique Afridi², Julio Carlos Pereira da Silva³, Bruna Andrade⁴, John Quensen⁵, James Tiedje⁵, Jorge Teodoro de Souza², Viviane Talamini⁶, Eudes de Arruda Carvalho⁷, Flávio Henrique Vasconcelos de Medeiros²

¹ Bayer Crop Science, Sorriso, MT, Brazil

² Department of Plant Pathology, Federal University of Lavras (UFLA), Lavras 37200-900, MG, Brazil

³ Universidade Federal de Santa Maria, Department of Plant Health, Santa Maria, RS, Brazil

⁴ Agrícola Andreis, Av. Barão do Rio Branco, 1597, Cascavel, PR, Brazil

⁵ Department of Plant and Soil Science, Michigan State University, 1066 Bogue St., Room A540, East Lansing, MI, USA

⁶ Embrapa Tabuleiros Costeiros, Av. Beira Mar, 3250, Aracaju, SE, Brazil

⁷ Embrapa Recursos Genéticos e Biotecnologia (Cenargen), Parque Estação Biológica, Brasília, DF, Brazil

**Corresponding authors:**	Rafaela Araújo Guimarães - [rafaela.guimaraes3@ufla.br](mailto:rafaela.guimaraes3@ufla.br)

Flávio Henrique Vasconcelos de Medeiros - flaviomedeiros@ufla.br

**Supplementary Information.**

**Supplementary Fig. S1**

The PCA biplot shows the separation of soil samples categorized as suppressive (blue) and non-suppressive (red) based on microbial community composition. The first two principal components (PC1 and PC2) explained 64.39% and 24.08% of the variance, respectively. Suppressive soils clustered distinctly from non-suppressive soils along PC1, suggesting differences in microbial profiles between the two groups. The ordination indicates that the genera *Trichoderma* spp. and *Bacillus* spp. are positively associated with suppressive soils, contributing significantly to the separation along PC1. In contrast, Pseudomonas was more associated with non-suppressive soils and oriented in the opposite direction along PC2.

Although the PCA of biological parameters did not reveal a statistically significant clustering between suppressive and non-suppressive soils (PERMANOVA, p = 0.164) (Supplementary Fig. S1), negative correlations were observed between bait colonization by C. paradoxa and the abundance of culturable fungi, Trichoderma spp., and Bacillus spp. (r = -0.72, p < 0.01), suggesting their potential role in pathogen suppression.

**Supplementary Fig. S1.** Principal Component Analysis (PCA) of soil biological properties associated with suppressive and non-suppressive soils in coconut production systems. The biplot displays the distribution of samples (dots) based on their chemical profiles, with red representing non-suppressive soils and blue representing suppressive soils. Arrows indicate the direction and magnitude of each chemical variable’s contribution to the principal components.

**Supplementary Fig. S2**

The most common and highly abundant phyla was reported *Actinobacteria*, *Proteobacteria*, *Firmicutes* and *Acidobacteria* andChloroflexi in both suppressive and conducive soil. Although, the the relative abundance of *Actinobacteria*, *Proteobacteria*, *Firmicutes* and *Acidobacteria* andChloroflexi observed higher in conducive soil in relation to conducive soil. The taxonomic profile of the suppressive soil revealed that Actinobacteria wasthe mostabundant in the suppressive soil corresponding to 28.6% and 22.4% in conducive. Otherrepresentativeswere*Chloroflexi*, *Planctomycetes*, *Verrucomicrobia*, *Bacteroidetes*, *Gemmatimonadetes*andWPS-1 (Supplementary Fig. S2).

**Supplementary Figure S2.** Relative abundance of the top 10 bacterial phyla in conducive and suppressive soils. The bar plot illustrates the taxonomic composition at the phylum level, showing differences in microbial community structure between treatments. Actinobacteria and Proteobacteria were the most dominant phyla in both soil types. Notably, suppressive soils exhibited higher relative abundance of Actinobacteria, while conducive soils showed greater representation of Proteobacteria. Other phyla, including Firmicutes, Acidobacteria, and Planctomycetes, were present at intermediate levels, with subtle differences across treatments.
